# Nanomedicine for cancer targeted therapy with autophagy regulation

**DOI:** 10.3389/fimmu.2023.1238827

**Published:** 2024-01-04

**Authors:** Ketai He, Mingkun Chen, Jiao Liu, Shufang Du, Changyu Ren, Jifa Zhang

**Affiliations:** ^1^ Department of Neurology, Joint Research Institution of Altitude Health, West China Hospital, Sichuan University, Chengdu, Sichuan, China; ^2^ West China School of Stomatology, Sichuan University, Sichuan, China; ^3^ Department of Pharmacy, Chengdu Fifth People’s Hospital, Sichuan, China

**Keywords:** nanomaterial, multi-functional, cancer, autophagy, autophagy inhibitor

## Abstract

Nanoparticles have unique physical and chemical properties and are currently widely used in disease diagnosis, drug delivery, and new drug development in biomedicine. In recent years, the role of nanomedical technology in cancer treatment has become increasingly obvious. Autophagy is a multi-step degradation process in cells and an important pathway for material and energy recovery. It is closely related to the occurrence and development of cancer. Because nanomaterials are highly targeted and biosafe, they can be used as carriers to deliver autophagy regulators; in addition to their favorable physicochemical properties, nanomaterials can be employed to carry autophagy inhibitors, reducing the breakdown of chemotherapy drugs by cancer cells and thereby enhancing the drug’s efficacy. Furthermore, certain nanomaterials can induce autophagy, triggering oxidative stress-mediated autophagy enhancement and cell apoptosis, thus constraining the progression of cancer cells.There are various types of nanoparticles, including liposomes, micelles, polymers, metal-based materials, and carbon-based materials. The majority of clinically applicable drugs are liposomes, though other materials are currently undergoing continuous optimization. This review begins with the roles of autophagy in tumor treatment, and then focuses on the application of nanomaterials with autophagy-regulating functions in tumor treatment.

## Graphical Abstract

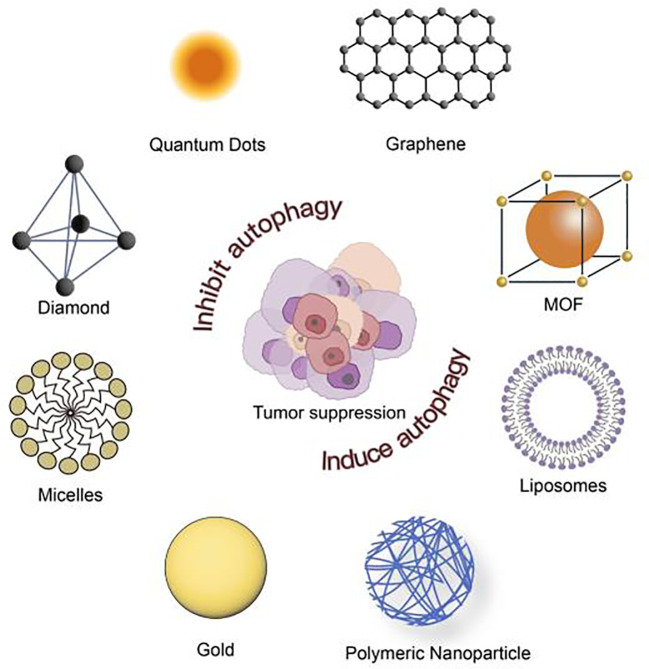

## 1 Introduction

Nanomaterials, including metal-based nanomaterials, carbon-based nanomaterials, lipid-based nanomaterials, and nano-polymer materials, have excellent physical or chemical characteristics such as controllable shape and size, large specific surface area, good biocompatibility, and easy surface modification (1). Particularly, some nanomedicines can improve drug pharmacokinetics and pharmacological properties, protect drugs from degradation in blood vessels, increase drug solubility, and deliver drugs in a tissue- or cell-specific manner, reducing drug accumulation in non-target organs and side effects (2). It appears to be successful to use nanoparticles as carriers for biomolecules, and some nanoparticles can influence numerous biochemical pathways and thus affect cellular metabolic activity. As a result, they are currently utilized extensively in biomedicine for a variety of purposes, including illness screening and diagnostics, drug delivery, the creation of novel drugs, etc.

Early cancer detection can result in prompt treatment and the best results, which contributes to much cheaper medical expenses and higher 5-year survival rates. Chemotherapy, when paired with surgery or radiotherapy, is the most crucial systemic therapy in the treatment of cancer because it increases the likelihood that local disease will be curable. Conventional chemotherapy can potentially harm normal cells and lacks a clear targeting mechanism. The patient’s quality of life will likely continue to decline and there is a chance that systemic toxicity will result (3). Nanotechnology’s development has given rise to hope for the early detection and treatment of cancer because it can improve the targeting capacity of anticancer drugs, boost local drug efficacy, thereby reducing systemic toxicity, improve diagnostic sensitivity, boost biological imaging, and improve radiotherapy (4).

The key material degradation system known as autophagy recycles big macromolecules or organelles to lysosomes where they are broken down into recycling-friendly tiny molecules like amino acids and monosaccharides. By removing defective or damaged organelles, autophagy serves a crucial role in preserving intracellular environmental homeostasis. Autophagy is closely linked to the occurrence and progression of diseases like cancer, diabetes, and neurological illnesses, according to recent studies (5). When compared to its involvement in other diseases, autophagy’s role in cancer is noticeably more complicated. It is thought to have a variety of functions depending on the stage of the disease; in the early stages of cancer, it mostly functions as an anti-cancer mechanism to slow the growth of the disease (6). It encourages the growth and spread of cancer in its advanced stages (via mechanisms such oxidative stress, nutritional restriction, or resistance) (7). As a result, controlling autophagy offers a potential means of preventing the growth of malignant tumors.

Several autophagy regulators face challenges, including poor solubility, non-specific distribution, and incompatibility with tumor acidic microenvironments (8). Using nanomaterials as drug carriers can enhance the therapeutic effect of the drug and reduce the occurrence of tumor resistance and chemotherapy side effects. In addition to acting as a nano-carrier, many nanoparticles themselves have autophagy-regulating effects, and specific material nanoparticles can also change multiple signaling molecule pathways in autophagy regulation. Therefore, research and development of nanomedicines for cancer targeted therapy with autophagy regulation may have great potential to provide ideas for the treatment of refractory and resistant cancers.

This article reviews the relationship between autophagy and tumors, the current application of drugs with autophagy effects in tumor treatment. Finally, we introduce the application of nanomaterials in tumor autophagy therapy and analyzes the development prospects of nanomedicines with important roles in regulating autophagy.

## 2 Autophagy and cancer

### 2.1 Autophagy regulatory mechanism

**Figure 1:**
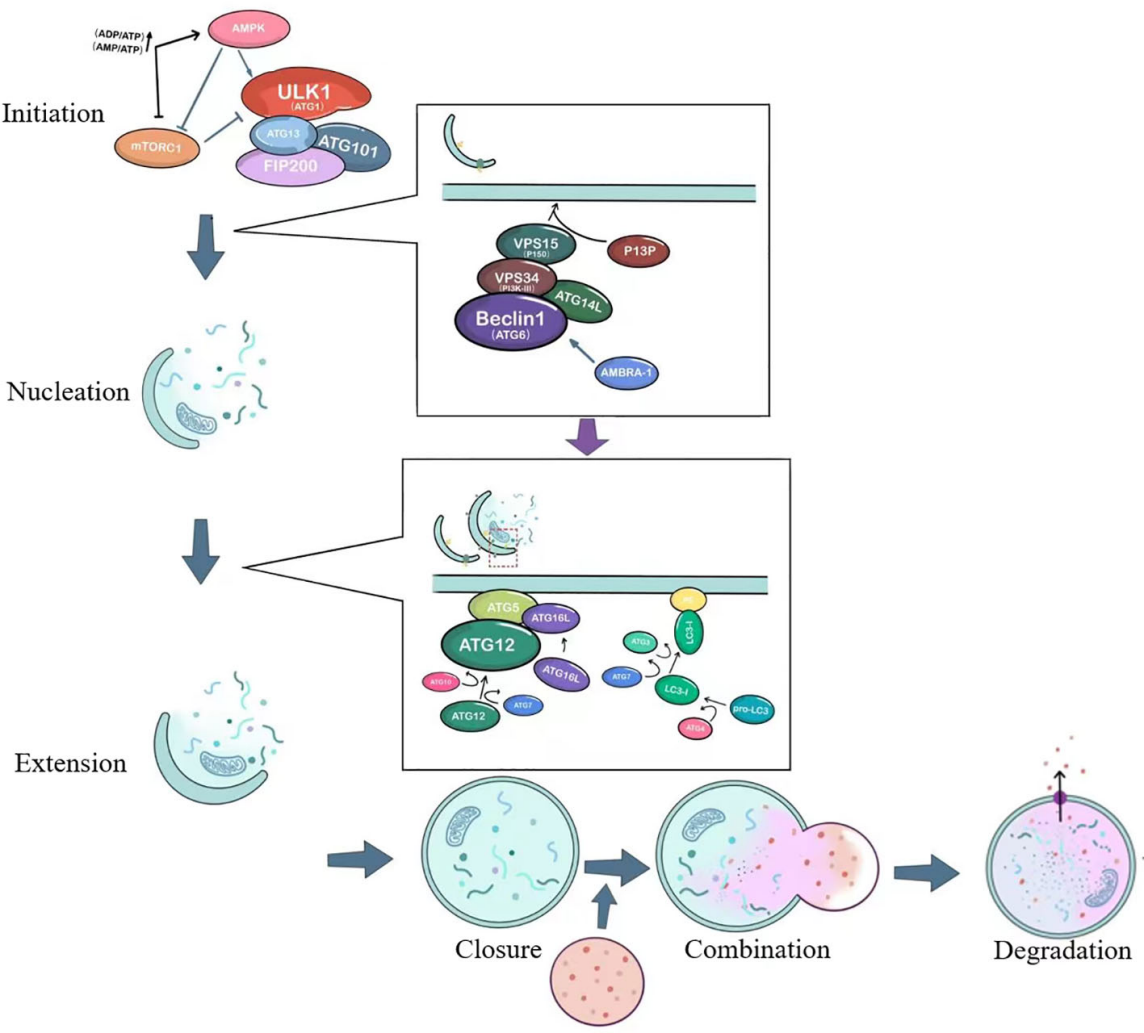
The basic process of autophagy. ULK1, unc-51-like kinase 1; mTORC1, Mammalian target of rapamycin complex 1; ATG, autophagy-related; AMPK, AMP-activated protein kinase; VPS34, vacuolar protein sorting 34; PI3P, Phosphatidylinositol 3-phosphate; AMBRA1, Activating molecule in Beclin-1-regulated autophagy.

It is currently generally believed that autophagy consists of six steps: initiation of autophagy, nucleation of autophagosomes, extension of autophagosome membranes, closure of autophagosome membranes, combination of autophagosomes with lysosomes, degradation and reuse of contents. The process and important targets of autophagy are shown in **Figure 1**.

The initiation of autophagy is closely related to the ULK1 complex, which includes the serine/threonine protein kinase ULK1, as well as ATG13, FIP200 (a protein that interacts with focal adhesion kinase FAK), and ATG101 (9), as shown in **Figure 1**. Under stable intracellular conditions, the mTORC1 complex binds to the ULK1 complex and phosphorylates ULK1 and ATG13, thereby inhibiting the initiation of autophagy (10). Under conditions of nutrient deprivation or starvation, AMPK responds to low ATP levels and can phosphorylate TSC2 (tuberous sclerosis protein), which enhances its inhibition of Rheb and suppresses mTORC1 activity (11). AMPK can also directly activate the ULK1 complex, thereby inducing autophagosome nucleation and elongation.

The ULK1 complex leads to the activation of the ATG6-VPS34 complex, which includes Beclin1 (ATG6), Bcl-2, VPS34, VPS15, ATG14L, and AMBRA-1 (12). This crucial complex is involved in the nucleation of the autophagosome, as illustrated in **Figure 1**. It generates phosphatidylinositol 3-phosphate (PI3P) on the cellular membrane in preparation for ensuing membrane curvature. Within the cytoplasm, cells form a small membranous structure called the isolation membrane, which extends continuously from both sides, flattens, and is referred to as the pre-autophagosome (PAS). The membrane originates from independent specialized membrane structures or organelles such as mitochondria, endoplasmic reticulum (ER), or Golgi complex, with the most common forming place at ER-mitochondria contact sites (13, 14).

The PAS interacts with the VPS34 complex under the action of two ubiquitin-like systems: the ATG12-ATG5-ATG16L conjugation system and the phosphatidylethanolamine/light chain 3 (PE/LC3) lipidation system, just as depicted in the second small box in **Figure 1**. This interaction extends the length of the membrane, which transforms from a crescent-shaped structure to a fully closed double-membrane structure called the autophagosome. The contents of the autophagosome consist of captured aged proteins, organelles, protein aggregates, and eventually become mature sealed autophagic vacuoles. Regulatory proteins such as Beclin1, ATG14L, AMBRA1, and ultraviolet radiation resistance-associated gene product (UVRAG) interact to modulate the extension of the double membrane (15). ATG12 is conjugated to ATG5 with the action of ATG10 and ATG7, and forms a complex with ATG16L. The precursor of LC3 is cleaved by ATG4 to generate a soluble form (LC3-I), which is then converted to a lipidated form (LC3-II) after the action of molecules such as ATG3 and ATG7 (16). LC3-II subsequently binds to the enriched lipid phosphatidylethanolamine (PE) in the newly formed autophagosomal membrane.

The autophagosome fuses with the lysosome, forming an autolysosome. This process is facilitated by proteins such as ATG15, ATG22, lysosome-associated membrane proteins (LAMPs), UVRAG, and the small GTPase Rab7 (12). UVRAG can form a complex with BECN1 and VPS34, regulating the formation of autophagolysosomes (17). The final step is the degradation and reuse of contents. Acid hydrolases inside the autolysosome degrade vesicle contents, which become degradative autolysosome contents. Degraded lysosomal enzymes release amino acids, monosaccharides, and other small molecules, which then exit through nutrient transport proteins for use in cell growth and development.

### 2.2 The role of autophagy in cancer

#### 2.2.1 Dual role of autophagy in cancer

**Figure 2:**
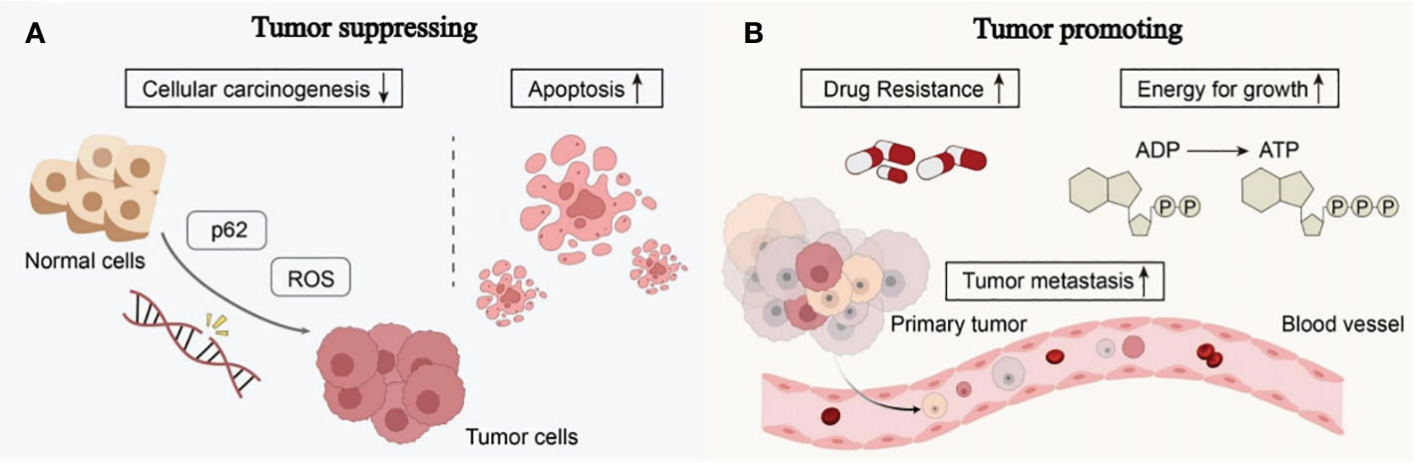
The complex role of autophagy in tumorigenesis: **(A)** Autophagy can reduce reactive oxygen species (ROS) levels and accumulation of p62, thereby reducing DNA damage and carcinogenesis in normal cells. Excessive autophagy can induce apoptosis in cancer cells, thereby inhibiting the occurrence and growth of cancer. **(B) **The complex role of autophagy in tumorigenesis involves providing nutrients and energy to tumor cells, inducing drug resistance in tumor cells, promoting tumor cell metastasis, and facilitating tumor development (see main text for details).

It is well known that many cancers have altered autophagy activity, and that autophagy can both promote and hinder the occurrence and progression of cancer. Some of the ways autophagy affects cancer are shown in **Figure 2**.

Autophagy works by eliminating toxic compounds within cells, maintaining metabolic equilibrium, and reducing DNA damage. When autophagy levels decrease, cells are unable to break down defective long-lived proteins and damaged organelles, leading to the accumulation of damaged proteins and the assembly of oncogenic p62 protein. Reactive oxygen species (ROS), which impact DNA sequence, structure, and function directly or indirectly, are produced in greater quantities in aging or damaged mitochondria that cannot effectively eliminate them (18). This results in DNA damage and genetic changes, ultimately leading to cancer. Excessive autophagy can induce autophagic death of tumor cells, thereby inhibiting cancer. Under continued stress and progressive autophagy, cells will die due to excessive self-consumption. The death of such cells often exhibits autophagic characteristics, manifested by overexpression of Beclin1 in the cells and the production of a large number of autophagosomes in the cells (19).

Autophagy can help tumor cells survive and multiply once they have formed. Cancer cells use autophagic mechanisms to combat nutritional deprivation and hypoxia when rapidly expanding tumor cells in solid tumors lack nutrients and have poor blood supply. Autophagy fuels the growth of tumor cells by destroying intracellular organelles and proteins, which helps them survive under stressful circumstances (20). Additionally, tumor resistance to therapeutic medications may be a result of cell-protective autophagy, which favors the survival of tumor cells. According to research, autophagy can influence antigen presentation by lowering MHC-I surface levels, which lowers anti-tumor T cell responses and fosters tumor growth (21). The ability of cancer cells to migrate and invade can also be improved by autophagy. It can strengthen resistance to anoikis apoptosis, accelerate the epithelial-mesenchymal transition in tumor cells, increase the release of pro-migratory cytokines like IL-6 through pathways involving TGF-β, and promote cancer cell invasion and metastasis (22).

#### 2.2.2 Autophagy regulating drugs

**Figure 3:**
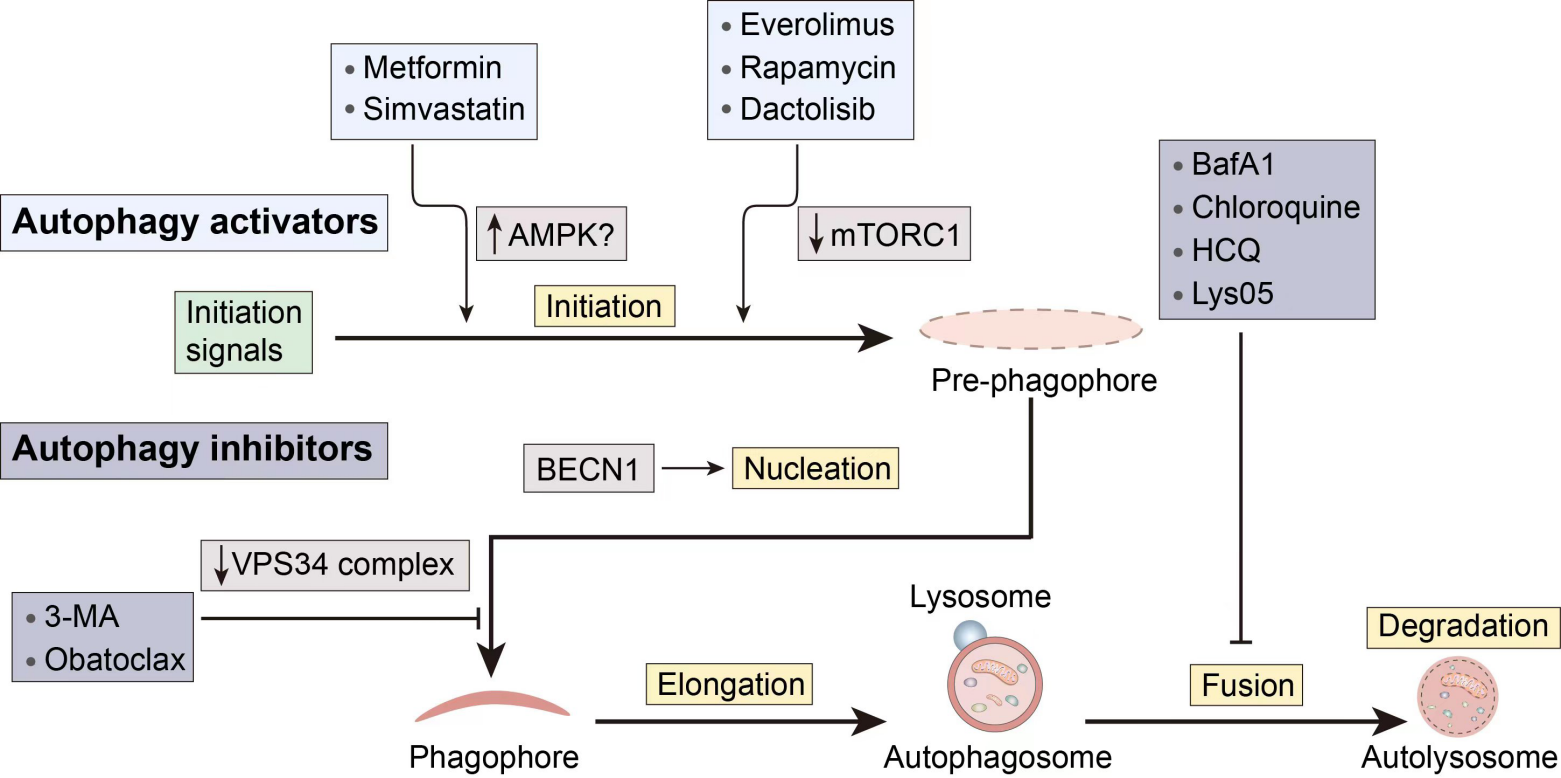
Several pharmacological interventions available to modulate autophagy. 3-MA, 3-methyladenine; BECN1, Beclin 1; CQ, Chloroquine; HCQ, hydroxychloroquine.

Given the complicated function that autophagy plays in cancer, autophagy modulators can be used to target autophagy and prevent the spread of cancer. For instance, the use of autophagy inducers in certain early-stage malignancies might cause apoptotic cell death, which can be used in cancer treatment. On the other side, autophagy aids in the survival and resilience of cancer cells to chemotherapy and targeted therapy. Therefore, the use of autophagy inhibitors is required to support the therapeutic benefits of chemotherapy and targeted therapy medicines. The stages of action and regulatory mechanisms of several autophagy modulators are shown in **Figure 3**.

### 2.3 Autophagy inducers

When there is an initiation problem, stimulating upstream autophagosome formation in tumor cells holds the potential to normalize autophagic flux (23). Furthermore, besides inducing apoptotic cell death in cancer cells with apoptosis resistance, autophagy can also act as an alternative cell death mechanism.

Metformin, a first-line drug for type 2 diabetes, has demonstrated a notable capacity to inhibit tumor growth. Its mechanism of action involves activating AMPK and suppressing mTOR, resulting in the inhibition of tumor development (24). Researchers have uncovered that metformin may trigger autophagic cell death, enhancing the susceptibility of sorafenib-resistant liver cancer cells (25). However, a phase 2 double-blind, randomized, and placebo-controlled trial (NCT01210911) indicated that individuals with advanced pancreatic cancer did not significantly benefit from taking metformin at the typical diabetic levels. The more potent biguanides may work better when coupled in tumor patients who are more sensitive to energy stress.

Another AMPK activator is simvastatin, which belongs to the statin class of drugs. Simvastatin has poor solubility in water and is currently used as an effective drug for treating high cholesterol and high triglyceride levels. Multiple reports have confirmed the anti-tumor potential of statin drugs in various cancers (26–28). The exact molecular mechanism by which simvastatin exerts its anticancer activity is not fully understood but may be related to AMPK activity regulation. Simvastatin stimulates autophagy, according to Wei et al., by activating mTOR, increasing LC3B and BECN1, and decreasing autophagosome-lysosome fusion (29). Furthermore, simvastatin’s anticancer effects may also be associated with its inhibition of Shh signaling, increased levels of RhoA-GTP, increased levels of HIF-1α, and induction of p38 MAPK activation etc. (30–32).

Rapamycin is a first-generation mTOR inhibitor and a macrocyclic lactone compound. Its derivatives (Rapalogs), including temsirolimus, everolimus, and ridaforolimus, have similar mechanisms of action, i.e., binding to the FRB domain of mTOR, altering the conformation of mTOR, and inhibiting mTORC1 kinase activity. Rapalogs have been approved as anti-cancer drugs (33), and the close relationship between the antitumor activity of rapamycin and its regulation of autophagy is evident. *In vitro* research by Lupinacci et al. revealed that rapamycin-induced autophagy mediated by p75NTR is a significant mechanism for tumor regression in Kaposi’s sarcoma (34). Due to the fact that rapamycin primarily induces autophagy through mTOR inhibition, when used in combination with an autophagy inhibitor whose mechanism involves inhibiting autophagosome fusion and degradation, its autophagy-inducing effect may be enhanced. Recent studies have also demonstrated this, showing that the combination of chloroquine and rapamycin synergistically targets autophagy, leading to an increase in the number of autophagosomes and apoptosis in 93T449 WDLS cells, thereby exerting a therapeutic effect on well-differentiated liposarcoma (35). A phase II clinical trial evaluating the efficacy of temsirolimus in patients with recurrent glioblastoma (NCT00016328) demonstrated reduced tumor-associated T2 hyperintensity on neuroimaging using magnetic resonance imaging (36). Despite the demonstrated inhibitory effects of rapamycin on various cancer cells, it still exhibits limitations in the treatment of drug-resistant cancers.

Dactolisib (BEZ235) is a hydrophobic synthetic imidazole and quinoline derivative, belonging to the second-generation mTOR inhibitors. It is an ATP-competitive inhibitor that binds to the ATP-binding site in the kinase domain of mTOR, thus simultaneously inhibiting mTORC1 and mTORC2, and partially inhibiting PI3K (37). Oral administration of highly selective PI3K/mTOR dual inhibitors may play a crucial role in synergistic cancer therapy. Helmy et al. demonstrated the significant impact of BEZ-235, when co-administered with Osimerin (an NF-κB inhibitor), in inhibiting the PI3K/Akt/mTOR/NF-κB axis, inducing apoptosis in HCT-116 colorectal cancer cells (38). Despite the favorable synergistic chemotherapeutic effects demonstrated by this combination in cancer treatment, the potential toxicity also underscores the impetus for further preclinical and clinical research involving lower concentrations of BEZ-235. BEZ-235 also exhibits the capability to induce autophagy and enhance the sensitivity of hepatocellular carcinoma cells to sorafenib by inhibiting PI3K/Akt/mTOR (39). Similar to the aforementioned rapamycin, it can be combined with chloroquine to achieve a more potent therapeutic effect than monotherapy (40). Furthermore, BEZ-235 demonstrates favorable therapeutic effects on cancers with mutations in autophagy-related targets. A study revealed that BEZ-235 may exert anti-tumor effects in mutant p53 (mutp53) triple-negative breast cancer cells by inducing autophagy. Evidence suggests a positive feedback loop between mutp53 and autophagy in these cells, further contributing to the anti-tumor effects (41). However, a phase I clinical trial evaluating the efficacy of Dactolisib and everolimus combination therapy in patients with advanced or metastatic solid tumors (NCT01508104) showed limited tolerability to the combination therapy, preventing dose escalation to potentially effective levels (42). Therefore, further exploration is needed to achieve high effectiveness.

Obatoclax is a BH3 mimetic and Beclin1 contains a BH3 structural domain, thus Obatoclax is able to bind competitively to Bcl-2 and act as an inhibitor of Bcl-2 family proteins (43). Sulkshane et al.’s study found that Obatoclax induces autophagy through the mechanism mentioned above, leading to autophagy-dependent necrotic apoptosis in human oral cancer cells. The downregulation of ATG5 significantly inhibited autophagy and blocked Obatoclax-induced cell death (44). Another study by McCoy et al. demonstrated that Obatoclax independently induces ATG7-dependent autophagy in small cell lung cancer (45). On the other hand, Obatoclax can also affect lysosomal acidification, blocking the fusion of lysosomes with autophagosomes and exerting an inhibitory effect on autophagy (46). In a study focused on thyroid cancer cells, Obatoclax directly caused necrosis, and its cytotoxicity depended on its accumulation in lysosomes rather than its interaction with Bcl-2 family members (47). Additionally, there is research exploring Obatoclax as an autophagy inhibitor to assist in cancer treatment dominated by nutrient deprivation, providing potential help in cutting off existing energy sources during starvation therapy (48).

### 2.4 Autophagy inhibitors

Autophagy plays a significant role in the pathogenesis of diseases. Inhibition of autophagy is anticipated to restore normal autophagic degradation and mediate the therapeutic effect or to increase the sensitivity of tumor cells to radiotherapy, when autophagy contributes mechanistically to the etiology of a disease or when autophagy can increase the resistance of tumor cells to radiotherapy at a specific stage.

3-methyladenine (3-MA) inhibits autophagy by blocking the formation of autophagosomes through the inhibition of type III PI3K, also known as VPS34. Research by Dong et al. demonstrated that autophagy may serve as a self-defense mechanism in colon cancer HCT116 cells during hypoxic treatment, and the combination of 3-MA with hypoxic treatment significantly suppressed hypoxia-induced autophagy, increased cell apoptosis, and exerted anticancer effects (49). Enhanced autophagy is associated with cisplatin resistance in cancer cells. Liu et al. utilized methods such as monodansylcadaverine staining and flow cytometry to demonstrate that the inhibition of autophagy by 3-MA augmented apoptosis in A549 cancer cells induced by cisplatin and paclitaxel (50). Another study found that, apart from inhibiting Beclin-1-mediated autophagy in pharyngeal squamous cell carcinoma, 3-MA also arrested the cell cycle, promoted apoptosis, and reversed cisplatin resistance (51). To date, 3-MA has not entered clinical trials.

Bafilomycin A1 (BafA1), Bafilomycin B1 (BafB1), Bafilomycin C1 (BafC1), and spautin-1 are inhibitors that hinder the fusion of autophagosomes with lysosomes. They are common V-ATPase inhibitors. V-ATPase is a multi-subunit transmembrane complex responsible for the acidification of lysosomes. Although not a direct target of autophagy, targeting V-ATPase can block autophagic flux. Researchers discovered that through fluorescence probes and protein blotting that BafA1, by inhibiting autophagy, increased the sensitivity of osteosarcoma cells to chemotherapy drugs (52).

CQ and HCQ are early and clinically used autophagy inhibitors. Originally developed as an antimalarial drug, it is currently applicable in clinical settings with relatively low cytotoxicity. In addition to inhibiting fusion, they can also prevent lysosomal acidification, thereby inhibiting the degradation of autophagic cargo. Furthermore, it has been shown that Lys 05 (a dimeric form of CQ) exhibits stronger autophagy inhibition than CQ or HCQ and demonstrates significant monotherapy anti-tumor activity without toxicity in mice treated with lower doses of Lys 05 (53). Numerous clinical trials with CQ and HCQ have been conducted for various types of cancer, including solid tumors, melanoma, sarcoma, glioblastoma, and breast cancer, among others (54).

## 3 Nanodrugs exert cancer therapeutic effects through autophagy regulation

**Figure 4:**
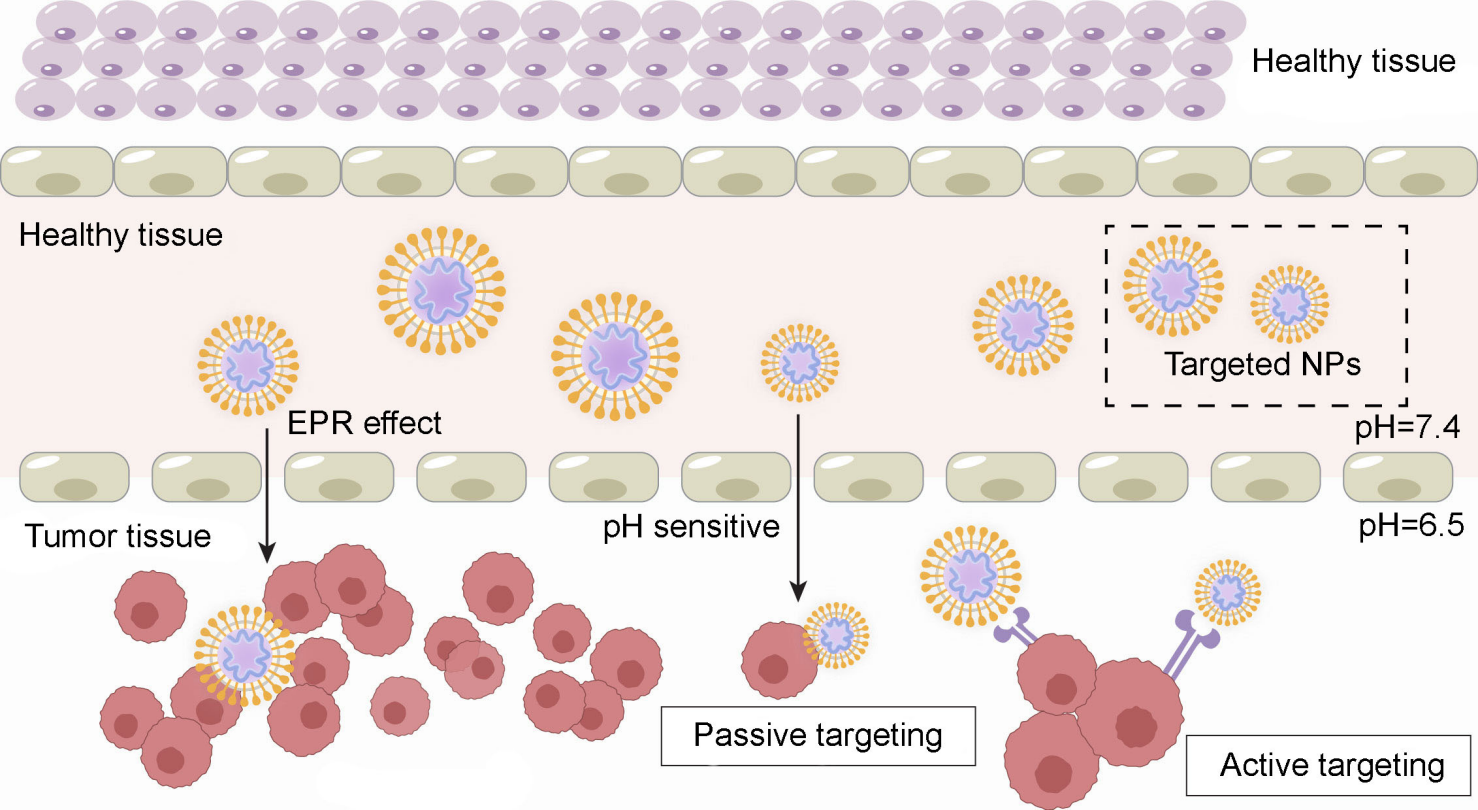
Passive and active tumor drug targeting.

Nanomaterials can also be specifically modified to possess high targeting specificity, delivery efficiency, and low systemic toxicity (55). Autophagy modulators face challenges such as limited solubility, nonspecific biological distribution, and restricted compatibility with the acidic tumor microenvironment (8). Utilizing nanomaterials for delivery presents a promising solution to overcome these issues. Cancer cells often exhibit higher sensitivity to certain nanoparticles than normal cells, and nanoparticles have great potential for passive tumor targeting. The high vascular permeability in tumors and the prolonged retention resulting from lymphatic obstruction also contribute to the specific accumulation of nanomedicines within tumor tissues. This phenomenon is known as the Enhanced Permeability and Retention effect (EPR). Additionally, nanoparticles (NPs) can enhance the solubility and stability of drugs in aqueous environments, extend their circulation time in the bloodstream, accumulate at the sites of tumor lesions, and increase the intracellular accumulation of anticancer compounds (56). This passive targeting effect on tumor tissues leads to increased therapeutic efficacy and reduced side effects. In addition, active targeting of cancer cells can be achieved by specific molecular modification or ligand functionalization of nanomaterials. **Figure 4** illustrates the passive and active targeting of nanoparticles to tumor cells.

**Figure 5:**
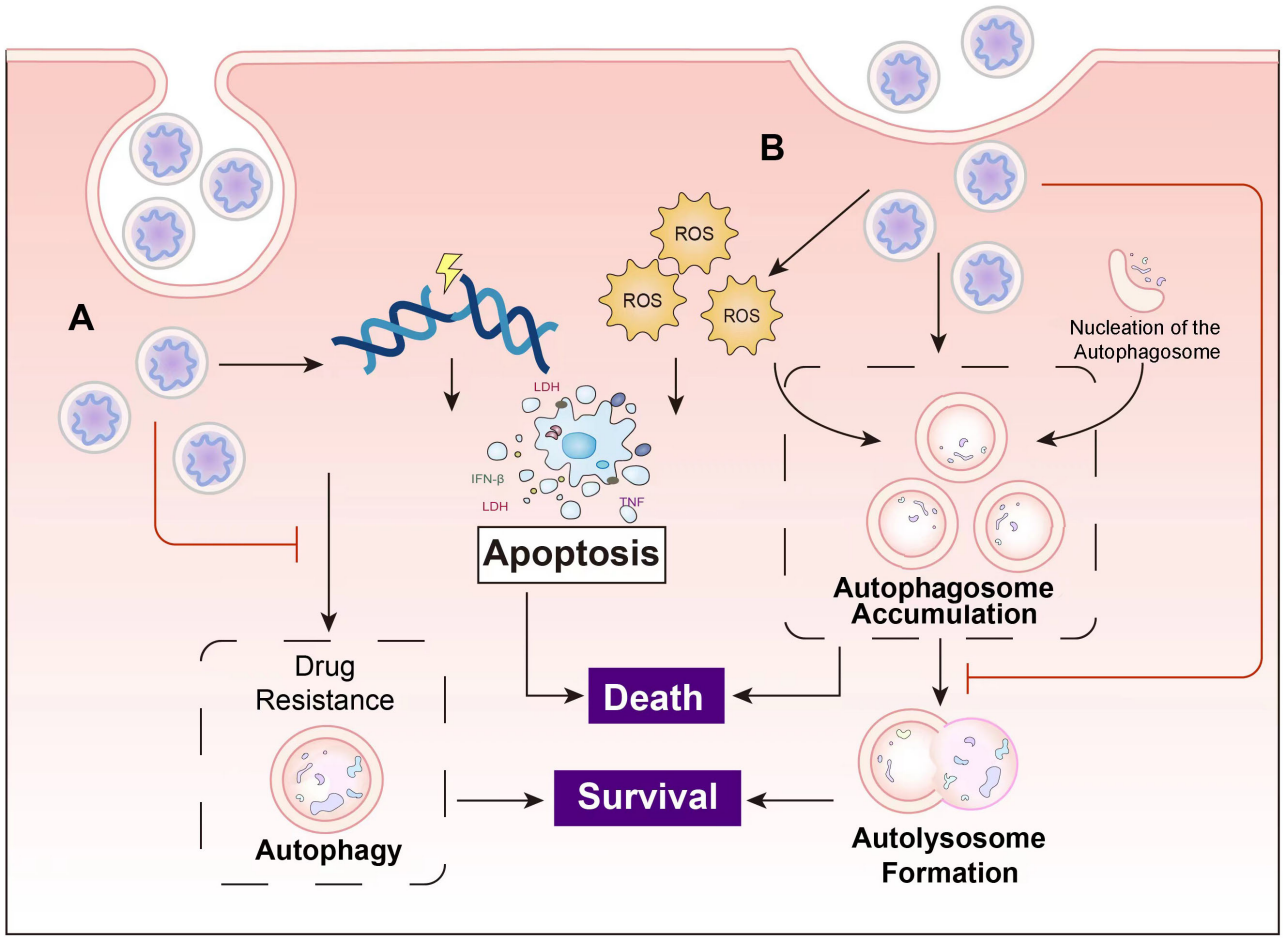
The main principles of the cancer inhibition of nanocomposites. **(A)** Nanocomposites induce apoptosis in cancer cells through mechanisms such as DNA damage, and overcome drug resistance by suppressing autophagy. **(B)** Some nanocomposites induce autophagy while concurrently inhibiting autolysosome formation, leading to autophagosome accumulation and ultimately resulting in cell death.

Moreover, beyond serving as drug carriers, nanoparticles (NPs) also act as foreign biomolecules within the body, falling within the size range of viruses and some small bacteria. Consequently, they possess inherent autophagy regulatory effects. **Table 1** provides a compilation of the diverse applications of different nanomaterials in cancer therapy through their autophagy regulatory effects. **Figure 5** illustrates the main principles of tumor cell inhibition through autophagy suppression or autophagy induction following the entry of nanocomposites into cells.

**Table 1:** Applications of nanomaterials related to autophagy regulation in cancer treatment.

Anti-cancer principle of nanocomposites	Nanomaterials	Cancer Type	In vitro/in vivo model	Size (nm)	Loaded drug/additional substances	Role of nanomaterials	Results	Ref.
Overcoming Tumor Drug Resistance by autophagy inhibition	Liposomes	Recurrent Ovarian Cancer	ES2-luc tumor-bearing mice	–	–	Inhibition of autophagy	Effective inhibition of autophagy/mitophagy, overcoming ovarian cancer drug resistance	(57)
	Liposomes	Breast Cancer	BxPC-3 cells, heterogenous pancreatic tumor model	130	HCQ	Enhanced targeting	Autophagy inhibition combined with cytotoxic drugs effectively treats dense stromal pancreatic cancer	(58)
	Micelles	Prostate Cancer	PC3 cells	129.9	Si-Berclin1, doxorubicin (DOX)	Tumor-targeted delivery	Combination of autophagy inhibition and chemotherapy in cancer treatment	(59)
	Gold nanorods	Glioblastoma	U-87 MG cells	142X42	Titanium dioxide	Inhibition of lysosomal protein, blocking autophagosome-lysosome fusion	Reduced autophagic flux, increased sensitivity to proteasome inhibitors-induced cell death	(60)
	Nanodiamond	Liver Cancer	HepG2 cells, In situ liver cancer transplantation mice	50-300	–	Damaged autolysosomal efflux	Assisted arsenic trioxide in solid tumors, showing potential for treating drug-resistant tumors	(61)
Assisting in radiotherapy by autophagy inhibition	Gold nanospikes	Oral Squamous Cell Carcinoma, Cervical Cancer	KB cells, U14 xenograft mouse model	54	–	Increased ROS production, mitochondrial depolarization, and redistribution of the cell cycle	Impact on endocytosis, radiotherapy,	(62)
Assisting in starvation therapy by autophagy inhibition	Metal-Organic Framework	Liver Cancer, Breast Cancer	HepG2 cells, 4T1 cells	100	CQ, Glucose Oxidase (GOx)	Induced ROS production; Repolarized M2 tumor-associated macrophages	Inhibited autophagy to enhance starvation therapy mediated by GOx	(63)
Directly inhibiting cancer cells by autophagy inhibition	Liposomes	Breast Cancer	4T1 tumor-bearing mice	120	HCQ, IGF2R-targeting peptide	Targeted delivery, increased sensitivity to HCQ	Autophagy inhibition, effective for cancer treatment	(64)
	Porous Cerium Dioxide Nanorods	Squamous Cell Carcinoma	SCL-1 cells	60X8X2	–	Catalyzed ROS clearance	Catalyzed autophagy inhibition and activated intrinsic antioxidant pathway in tumor cells	(65)
	Graphene Quantum Dots	Liver Cancer	HepG2 cells	17.62	Mn	Maintained high photodynamic efficiency in lysosomal environment; ROS generated by light primarily affected lysosomal function, resulting in lysosomal damage and effectively blocking protective autophagic recycling	Sustained increase in oxidative stress levels in lysosomes led to severe dysfunction of autophagy, as indicated by the abnormal increase in autophagosomes and autolysosomes. This ultimately resulted in autophagy-related cancer cell death accompanied by apoptosis and iron deposition.	(66)
Assisting in immunotherapy by autophagy inhibition	Metal-Organic Framework	Melanoma, Lung Metastasis	B16F10 cells, Lung Metastasis Mice	296	CQ	Induced autophagy by recruiting LC3 protein	Successfully inhibited over 90% of lung tumor lesions	(67)
Inducing apoptosis related to cellular autophagy	Liposomes	Breast Cancer	4T1 cells	135	HCQ, anti-PDL1 antibody	Mimicking viral lysosomal escape and autophagy induction	Upregulation of autophagy, inhibition of autophagic flux, leading to tumor cell apoptosis	(68)
	Micelles	Ovarian Cancer	SKOV3, A2780, OVCAR3, OVCAR8, SNU119, OVSAHO cells	50	–	Induction of HR gene downregulation, affecting cell proliferation, migration, apoptosis, and autophagy	Combined with PARP inhibitors, induction of HR repair defects in cancer cells	(69)
	Gold nanoparticles	Triple Negative Breast Cancer	TNBC cells, xenograft mouse model	10X40	Anti-EGFR antibody	Formation of autophagic vesicles, inhibition of Akt-mTOR signaling pathway	Significant induction of autophagy, leading to targeted cell death in EGFR-positive cancer cells	(70)
	Zinc Oxide	Liver Cancer	Huh7 cells	14.13	–	Upregulated p53 gene, induced autophagy	Induced autophagy and promoted apoptosis in liver cancer cells	(71)
	Graphene Oxide	Colorectal Cancer	HCT116 cells, Xenograft model mice	0.67	–	ROS-dependent AMPK/mTOR/ULK-1 pathway	Induced autophagic death in HCT116 cells, exerting anti-colorectal cancer effects	(72)
Optimizing Autophagy and Immunomodulatory Effects of Rapamycin-Class Drugs	Micelles	Breast Cancer	MCF-7 breast cancer cells, ascitic tumor-bearing mice	212.2	Rapamycin, hanhuangchinoside	Enhanced tumor targeting, prolonged nanoparticle circulation; reduced particle size and improved stability	Inhibition of tumor growth, improved anti-cancer efficacy, reduced side effects	(73)
	Polymer nanoparticles	Breast Cancer	MCF-7, SKBR3 cells	101.3	Everolimus	Active targeting to SKBR3 cells (HER2+)	Synergistic effect in inhibiting breast cancer cell growth and reducing cell viability	(74)
	PLGA-PCL nanoparticles	Breast Cancer	MCF-7, Jurkat cells	0.1	Rapamycin	Inhibition of cell proliferation, maintaining bioactivity of rapamycin	Enhanced anti-cancer effect compared to free rapamycin	(75)
Increasing Autophagic Flux to Reduce Drug Dosage and Toxicity	Magneto-gold@fluorescent polymer	Liver Cancer	HepG2 cells, HepG2 xenograft mice	30	–	Facilitated autophagic degradation	Enhanced autophagosome formation without interfering with autophagosome-lysosome fusion, exhibiting improved chemosensitization ability	(76)
Activating the Immunogenicity of Tumor Cells through Autophagy Induction	Nano-DOX	glioblastoma	U87 MG cells	83.9 ± 32.3	Doxorubicin	Induce autophagy in GBM cells and stimulate it to emit antigens and damage-associated molecular patterns, resulting in enhanced activation of dendritic cells.	Doxorubicin-polyglycerol-nanodiamond composites could activate autophagy in GBM cells and thereby stimulate the immunogenecity of GBM cells.	(77)

### 3.1 Liposomes

Liposomes, composed of one or multiple layers of phospholipid molecules, are one of the earliest drug nanocarriers used in cancer therapy (78). Liposomes can be modified on their surfaces to serve as environment-sensitive delivery systems or actively target specific cells through ligand conjugation (79). Due to the phospholipid bilayer’s similarity to biological membranes, drugs encapsulated in liposomes readily interact with cells. This imparts excellent biocompatibility and biodegradability to liposomes, effectively improving drug solubility and enhancing drug targeting.

In cases where cancer is closely associated with autophagy, nanomedicines loaded solely with autophagy modulators can achieve significant therapeutic efficacy. Currently, liposome-based nanocarriers approved by the United States Food and Drug Administration (FDA) are being used for cancer treatment (80). Research has demonstrated that liposomes loaded with rapamycin, prepared using a film hydration method and pre-loading technique, can achieve better mTOR inhibition to promote autophagy. This approach significantly suppresses bladder cancer tumor cell growth while reducing the drug’s side effects on other cells (81). In this study, folate-modified liposomes, due to folate receptor-mediated endocytosis, were shown to be taken up by cells more than twice as effectively, providing an important avenue for improving liposome targeting. As mentioned earlier, rapamycin generally exhibits limited efficacy in the treatment of drug-resistant cancers.

Inhibiting autophagy can effectively improve the treatment outcomes of drug-resistant cancers, as cancer cell resistance is frequently associated with autophagy. The mechanisms underlying drug resistance can be multifaceted, as cancer cells often enhance autophagy to reduce drug accumulation within cells, thereby mitigating the toxic effects (82). Moreover, in pancreatic ductal adenocarcinoma, autophagy promotes collagen production, leading to increased stromal density, which severely hampers the effectiveness of anticancer drugs (58). Chen et al. loaded hydroxychloroquine onto liposomes, which demonstrated excellent targeting and penetration effects *in vitro* and *in vivo*. This approach effectively inhibited autophagy in pancreatic cancer cells and adjacent cancer-associated fibroblasts, enhancing paclitaxel’s cytotoxicity against pancreatic cancer cells (58). Given the overexpression of integrin αvβ3 receptors on the surface of cancer cells and the acidic microenvironment in their vicinity, the design of integrin αvβ3-targeted and pH-sensitive liposomes in this study enhances the specificity of nanomedicines, offering intriguing prospects in the field of nanodrug design.

Inhibition of autophagy can potentially aid in the treatment of drug-resistant cancers, while excessive autophagy induction can also trigger autophagic cell death. Kang and colleagues developed a tumor-targeted co-delivery system by encapsulating dihydroartemisinin and DOX in mannosylated liposomes. This approach increased the accumulation of therapeutic agents in the nucleus, enhanced apoptosis in cancer cells, and exerted an inhibitory effect on drug-resistant colon cancer cells (83). Researchers developed the nano-drug 188Re-liposome, which can inhibit autophagy and mitophagy in ovarian cancer, thereby reversing drug resistance and resulting in significant tumor suppression (57). Even at the first-level dose in Phase I clinical trials, it showed promising results, suggesting a potential new strategy to overcome drug resistance in ovarian cancer. Another study loaded dihydroartemisinin and DOX onto liposomes and found that these nanoparticles, in addition to taking advantage of the EPR effect, downregulated the activity of Bcl-2 and accelerated its dissociation from BECN1, leading to excessive autophagy induced by BECN1 and type II programmed cell death in breast cancer cells. This approach enhanced the therapeutic efficacy against breast cancer (84). This research highlights that liposomes, while enhancing their targeting capabilities and extending drug circulation, also exhibit autophagy induction on their own.

Therefore, by properly modulating autophagy, it is possible to alter the response of drug-resistant cells to anticancer drugs, increase their sensitivity to drugs, and ultimately achieve better treatment outcomes. Furthermore, pH-sensitive liposomes modified with hydroxychloroquine can inhibit lysosomal acidification and prevent autophagosome degradation. Phase II clinical trials with these liposomes have also been completed (NCT00726596, NCT04841148).

### 3.2 Micelles

Micelles are tiny spherical particles composed of one or multiple layers of surfactant molecules, typically consisting of a hydrophilic shell and a hydrophobic core. The hydrophobic core can load poorly water-soluble hydrophobic drugs, while the hydrophilic shell can prolong the drug’s circulation time in the body (85). Nanomicelles exhibit high optical transparency, excellent stability, small particle size, and a narrow size distribution, along with good biocompatibility and low toxicity (86). Micelles can also be surface-modified and offer ample structural adjustment space (73). The drug release characteristics of micelles depend on the nature and structure of the surfactants, typically involving drug release through surface diffusion or micelle disruption (87).

Nanomicelles can also be used to deliver rapamycin and directly inhibit cancer growth through autophagy regulation. Rapamycin is a commonly used autophagy inducer and has been developed as a potential anticancer drug for rapamycin-sensitive cancer models. However, its poor water solubility greatly hinders its application in cancer treatment. Chen et al. used dual-responsive mixed micelles to provide an appropriate delivery system for poorly water-soluble drugs like rapamycin in gastrointestinal oral administration for cancer treatment. They found that rapamycin dual-responsive micelles exhibited excellent antitumor activity and a high apoptosis rate in HCT116 cancer cells (88). Another study used self-assembled amphiphilic zein-ferritin micelles for the co-delivery of rapamycin and wogonin to target breast cancer tumors. These micelles demonstrated relatively rapid wogonin release and slow rapamycin release, facilitating the inhibition of efflux pumps by wogonin. This made cancer cells more sensitive to rapamycin, allowing for reduced drug dosages while minimizing side effects and maximizing antitumor efficacy against breast cancer (73). Another study co-delivered rapamycin with the anticancer drug 9-nitro-20(S)-camptothecin (9-NC). Rapamycin induced autophagy in tumor cells and enhanced their sensitivity, while surface-modified micelles directly targeted delivery to the cell nucleus, resulting in significant anticancer effects (89).

Similar to nanoliposomes, nanomicelles themselves can possess autophagy-inducing capabilities. While serving as drug carriers, they may induce intracellular autophagy, potentially affecting the action of loaded drugs (90). Therefore, autophagy inhibitors are often co-delivered with these cancer treatment drugs to assist in the therapeutic process (91). Zhang et al. prepared polymer micelles using a polymer membrane dialysis method and found that these micelles indeed induced intracellular autophagy, aggravating drug resistance. However, PEG-PLGA micelles loaded with docetaxel and chloroquine could effectively suppress autophagy in MCF-7 cells, enhancing their antitumor activity (90). Another study used cholesterol-penetratin self-assembled micelles as a drug delivery system, discovering that cationic cholesterol-penetratin micelles loaded with miR-124 could effectively induce autophagy, leading to the degradation of miR-124 in the corresponding autophagic lysosomes (92). However, when BH3 mimetic obatoclax (as mentioned earlier) was encapsulated, it inhibited the degradation of miR-124 and p62 in autophagic lysosomes, thereby maintaining the concentration of miR-124 in breast cancer cells. This enhanced the cytotoxicity of miR-124 against breast cancer cells. Cholesterol-penetratin is a classical cell-penetrating peptide that can aid in drug transmembrane delivery, providing important insights for subsequent synthesis and modification of nanocarrier micelles.

Another study adopted a similar strategy but made improvements to the micelle material. Rao et al. prepared nanomicelles for co-delivery of the chemotherapeutic drug DOX and the autophagy inhibitor wortmannin using a Cu(I)-catalyzed click chemistry-triggered azide/alkyne aggregation system. These nanomicelles exhibited excellent antitumor activity against melanoma and breast cancer (87). The size of these micelles increased in a time-dependent manner, offering better penetration and accumulation than fixed-sized nanoparticles (NPs), which enhanced their therapeutic efficacy against cancer cells. In another study, the nanomicelles did not have a fixed particle size. This research designed an *in-situ* self-assembling nanomicelle-dissolving microneedle (DMN) patch, co-encapsulating the immunogenic cell death inducer IR780 and CQ-loaded micelles (C/I-Mil). Photo-thermally mediated size-adjustable C/I-Mil effectively penetrated deep into tumor tissues, achieving highly targeted delivery to the deep tumor tissue through endocytosis and autophagy inhibition. This approach effectively reshaped the tumor’s immunosuppressive microenvironment, ultimately eliminating primary and distant tumors (93).

Apart from preventing the development of drug resistance, minimizing side effects is also a crucial consideration in the selection and preparation of nanomaterials. González-Pastor et al. encapsulated cisplatin and CQ using Pluronic F127 hybrid dendritic-linear-dendritic block copolymer micelles. Their research found that micelles loaded with cisplatin and chloroquine increased cytotoxicity in tumor cells while maintaining a lower level of cytotoxicity in non-tumor cells (94).

Currently, there are not many clinical trials involving micelles. The main focus has been on clinical trials involving cisplatin and docetaxel, where researchers have been studying the pharmacokinetics, safety, and tumor efficacy (NCT05478785, NCT05254665, NCT01023347). Once these clinical trials yield positive results, there may be future clinical trials involving micelles loaded with autophagy inhibitors to combat multidrug resistance.

### 3.3 Polymers

Polymer nanoparticles (PNPs) are nanocarriers composed of high-molecular-weight compounds. They possess good stability, highly controlled drug release, desirable pharmacokinetics, and potential for biomedical imaging and photodynamic therapy (95). Some polymer nanoparticles can achieve controlled release delivery by changing their structure or responding to external stimuli, such as drug release triggered by ultrasound, which are known as stimulus-responsive nanoparticles (96). Currently, most nanopolymer research and use focus on biodegradable polymers such as polylactic acid (PLA), poly(lactic-co-glycolic acid) (PLGA), polyamino acids, and alginate, which offer good biocompatibility (97).

Nanopolymer carriers are often used to deliver autophagy inhibitors to counteract chemotherapy resistance induced by autophagy-promoting tumorigenesis. Zhang et al. found that PLGA nanoparticles could trigger autophagy in tumor cells, potentially affecting the efficacy of docetaxel treatment. However, incorporating autophagy inhibitors such as 3-MA and CQ within the nanoparticles significantly improved the therapeutic efficacy of the nanoparticle formulation (98). In another experiment, DOX and the autophagy inhibitor LY294002 were encapsulated in pH-sensitive hydrophilic polymer nanoparticles prepared from hyperbranched polyacylhydrazone (HPAH). Upon penetration into oral cancer cells, drug release was responsive to acidic pH values, and both worked together to inhibit cancer progression (99). In these nanoparticles, the hydrophobic DOX was linked to the hydrophilic HPAH, forming micelles and loading the autophagy inhibitor. This ensured the preferential release of LY294002, inhibiting autophagy in tumor cells, making them more sensitive to the subsequent release of DOX. This design of amphiphilic micelles exhibits good targeting and therapeutic effects, and the sequence of release has a significant impact on subsequent drug loading.

PNPs can also be induced through autophagy, showing enhanced cytotoxicity, especially in autophagy-sensitive tumor cells. Wang et al. prepared PNPs loaded with BECN1. The complex showed enhanced cytotoxicity to breast cancer cells by inducing autophagy, and the PNPs themselves also had a certain autophagy-inducing effect (100). Autophagy induction can also be used for effective targeted photothermal therapy. Another study used BECN1-derived peptides for tumor targeting and autophagy-promoting photothermal therapy, and found that it upregulated autophagy in cancer cells and further sensitized tumors to photothermal ablation (101). Especially at high concentrations, polymer nanoparticles are more likely to induce autophagic cell death (102).

In the context of polymer nanoparticles, there are currently relatively few clinical trials related to autophagy regulation in cancer treatment. However, there is an upcoming preclinical trial (NCT05456022) that will explore the close relationship between quercetin and its encapsulated nanoparticles in the treatment of oral squamous cell carcinoma, with a focus on cellular autophagy.

### 3.4 Metal-based materials

Metal-based nanoparticles are nanoscale particles composed of metal elements or metal compounds, which exhibit unique physical, chemical, and biological properties, showing significant cytotoxicity in a time-dependent and dose-dependent manner in cancer cells (103). Common examples include metal oxide nanoparticles, metal-organic frameworks (MOFs), and gold nanoparticles. Metal-based nanoparticles can regulate autophagy through mechanisms such as oxidative stress, Akt/AMPK/mTOR pathway dysregulation, mitochondrial damage, and ER stress (104). Compared with autophagy inducers, there are few inhibitory nanoparticles like gold nanospikes.

Gold nanoparticles offer a high degree of tunability in terms of size and shape, and their surfaces can be easily functionalized with chemotherapy drugs, antibodies, or nucleic acids, while maintaining good biocompatibility and biocompatibility, making them excellent inducers of autophagy for cancer treatment. Surface modifications can be performed using polyethylene glycol (PEG) or specific antibodies to enhance the targeting of gold nanoparticles to tumors, reduce toxicity to other tissues, and significantly increase the cellular uptake of nanodrugs (70). Furthermore, gold nanoparticles as carriers can mitigate the progression and vitality of breast cancer by providing photothermal therapy while also exhibiting bioimaging capabilities (105). Gold nanoparticles induce excessive ROS production, subsequently triggering apoptosis and autophagy, thereby exerting a killing effect on cancer cells (106). A study shows that the mechanism of coated gold nanobipyramids inhibiting autophagy in glioblastoma cells is mainly achieved by inhibiting lysosomal proteins and thereby blocking the autophagosome-lysosome fusion process (60). Another study showed that gold nanospike can inhibit the progression of oral epidermal cancer and cervical cancer through the increase of ROS, mitochondrial depolarization and cell cycle redistribution (62). Currently, there are preclinical and pilot human clinical studies involving gold nanoparticles in the treatment of breast cancer (107). The role of autophagy in these studies is still under investigation and requires further research to better understand its involvement and potential impact on the treatment outcomes.

Metal oxide nanoparticles are another commonly used nanomaterial, encompassing various types such as zinc oxide, iron oxide, copper oxide, and cerium oxide. Some nanoscale oxide materials exhibit unique effects in cancer treatment due to their specific physical properties. Superparamagnetic iron oxide nanoparticles (SPIONs) possess magnetic properties and can serve as drug carriers to address issues related to hydrophobic anticancer drugs. They exhibit excellent chemosens ity and, simultaneously, enable bioimaging. Moreover, they effectively induce autophagy, thus enhancing the sensitization of chemotherapy (76). On the other hand, porous cerium dioxide nanorods possess unique physical properties that catalytically scavenge ROS, inhibit autophagy, and activate the intrinsic antioxidant pathway of tumor cells, thereby suppressing squamous cell carcinoma of the skin (65).

Metal-organic frameworks (MOFs) are crystalline materials formed by orderly assembling organic linkers between metal nodes. These MOF materials exhibit distinctive characteristics, including a large surface area, high porosity, and tunable chemistry (108). Zeolitic imidazolate frameworks (ZIF-8), a prominent subclass of MOF nanoparticles, possess high loading capacity and pH-sensitive degradation capability. A study by chen et al. encapsulated 3-MA, a chemotherapy agent, within ZIF-8 nanoparticles and observed significantly inhibited autophagosome formation compared to free 3-MA, resulting in higher anticancer efficacy (109). Another study encapsulated CQ within ZIF-8 and further modified it with methoxy polyethylene glycol-folic acid (FA-PEG) for active targeting, which effectively disrupted autophagosome formation and autophagic flux, exerting therapeutic effects (110).

Overall, metal-based nanoparticles hold great promise for cancer treatment. Their unique physical and chemical properties, combined with tunable size, shape, and surface modifications, enable them to serve as drug carriers, inducers or inhibitors of autophagy, and therapeutic agents in various cancer types. Further research and development in this field will continue to expand their potential in precision medicine and targeted cancer therapy.

### 3.5 Carbon-based materials

Carbon exists in various allotropes, and its nanomaterials exhibit diverse forms, ranging from one-dimensional to three-dimensional structures, such as carbon nanotubes and carbon dots (CDs), graphene, and diamond.

CDs, belonging to quantum dots, have sizes typically smaller than 10 nm and possess excellent biocompatibility, safety, and fluorescent activity. CDs can be utilized in cancer treatment through photothermal therapy and induction of mitochondrial stress (111, 112). Due to their small size, quantum dots often induce autophagy, which is one of the pathways for inhibiting cancer progression. Bajpai et al. found that nitrogen-phosphorus-doped carbon dots can increase the expression of ATG5 and LC3-II in B16F10 melanoma cells, reduce the expression of p62, thereby inducing autophagy and apoptosis in melanoma cells (113).

Graphene, with its honeycomb structure, possesses high binding affinity and can be functionalized with various polymers to enhance active targeting of cancer cells. Graphene nanostructures can lead to autophagy and osteosarcoma inhibition by regulating various molecular pathways of autophagy such as upregulating ATG5, ATG7, and LC3-II (114). Like CD, graphene nanoparticles also have photothermal properties, which inhibit autophagy and conduct thermal stress, promoting apoptosis (115). When graphene nanoparticles are loaded with autophagy inhibitors, they can also exert an autophagy inhibitory effect by blocking autophagy flux and inducing the accumulation of autophagosomes in cells (116). Graphene oxide can also induce autophagic death of HCT116 cells by activating the ROS-dependent AMPK/mTOR/ULK1 pathway, thereby exerting obvious anti-colorectal cancer effects both *in vivo* and *in vitro* (72). And it can also synergize with cisplatin to significantly enhance the initiation and progression of autophagy in Skov-3 cells, enhance the toxicity of cisplatin to colon cancer cells, and mediate cell necrosis (117). Hybrid nanocomposites of graphene and silver nanoparticles can also have a synergistic effect with cisplatin on cancer inhibition, increasing the production of ROS in cervical tumors, thereby stimulating apoptosis and autophagy (117).

Diamond is another allotrope of carbon. High levels of autophagy under hypoxic conditions are an adaptive strategy for cancer cell survival, and nanodiamonds have been shown to synergistically inhibit autophagy under hypoxic conditions in tumors, thereby improving the arsenical-based therapy of solid tumors (61). This complex, Nano-DOX, stimulates the immunogenicity of glioblastoma cells by inducing autophagy and initiates anti-glioblastoma immune responses, which can effectively regulate its immunosuppressive microenvironment (77).

## 4 Summary and prospects

In this review, we summarized different types of nanocarriers that can be used to deliver autophagy regulators, including liposomes, micelles, polymers, carbon-based nanocarriers and metal-based nanocarriers, some of which have autophagy regulatory effects themselves. We also discussed various autophagy regulators loaded onto these nanocarriers and their targeting mechanisms.

Researchers have employed various strategies to enhance the efficacy of nanomaterials, including surface modification, pH sensitivity, adjustable size, and encapsulation of hydrophobic substances. These improvements have increased the materials’ targeting capabilities while reducing side effects and drug dosages. The underlying principles of using nanomaterials in cancer therapy through autophagy regulation encompass inducing autophagy in sensitive cells to inhibit tumor growth, suppressing autophagy to enhance the effectiveness of chemotherapy drugs, immunogenic cell death inducers, photothermal therapy, and inducing ROS to promote excessive autophagy and subsequent apoptosis in cancer cells.

The development of targeted anti-cancer nanomedicines with autophagy regulation has made significant progress, showing great potential in cancer treatment. Current research has gradually deepened and some drugs have entered phase I or II clinical trials. Despite decades of tremendous effort, the results achieved in technology/knowledge transfer have been limited so far. To date, the FDA has only approved five nanomedicines for the treatment of solid tumors.

Despite significant progress in this field, there are still some challenges to be addressed. One major challenge is to optimize the characteristics of nanocarriers such as size, surface charge and targeting ligands to improve their tumor targeting and cellular uptake efficiency. Another challenge is the lack of standardization and stability in the preparation and production processes of nanomaterials, and the heterogeneity of patient conditions and etiologies can also impact experimental results. Additionally, some nanomaterials may pose risks to human health and require comprehensive assessment and monitoring in clinical applications. Furthermore, the therapeutic effectiveness of certain nanomaterials may be incomplete, failing to meet clinical requirements.

Overall, different anti-cancer drugs have different physicochemical, pharmacokinetic and pharmacodynamic properties that determine their unique clinical efficacy and safety characteristics in human cancer patients. The development of targeted anti-cancer nanomedicines with autophagy regulation is a promising approach to cancer treatment. Additionally, different nano-delivery platforms should be developed for various drugs with a focus on tumor specificity due to the tissue specificity of many tumors. To enable nanomedicines with autophagy to enter clinical practice as soon as possible, future research directions may include reducing drug toxicity, stabilizing manufacturing methods, improving tumor inhibition efficiency, and lowering production costs. As nanocarrier properties continue to be optimized and more effective autophagy modulators are developed, these nanomedicines may provide more effective and personalized cancer treatment options.

## Author contributions

JZ conceived and designed this manuscript. CR participated in the writing of some sections of the paper and provided suggestions for revisions after the completion of the paper. SD assisted in the modifications following the paper's review, made changes to figures, and conducted the final review. KH created the figures and wrote the majority of the paper. JL and MC summarized the literature. All authors contributed to this article and approved the final version.
